# Bioinspired Design and Applications of Liquid Gating Gas Valve Membranes

**DOI:** 10.3390/biomimetics10020077

**Published:** 2025-01-26

**Authors:** Yiyao Li, Yang Liu, Rui Xu, Jing Liu, Xu Hou

**Affiliations:** 1 Research Institute for Biomimetics and Soft Matter, Fujian Provincial Key Laboratory for Soft Functional Materials Research, College of Physical Science and Technology, Xiamen University, Xiamen 361005, China; yiyaoli@stu.xmu.edu.cn; 2 State Key Laboratory of Physical Chemistry of Solid Surfaces, College of Chemistry and Chemical Engineering, Xiamen University, Xiamen 361005, China; lyyyq0118@stu.xmu.edu.cn (Y.L.); sharaexu@stu.xmu.edu.cn (R.X.); 3 Innovation Laboratory for Sciences and Technologies of Energy Materials of Fujian Province (IKKEM), Xiamen 361005, China; 4 Institute of Artificial Intelligence, Xiamen University, Xiamen 361005, China

**Keywords:** bioinspired design, gas valves, liquid gating membranes, stimuli response

## Abstract

In nature, dynamic liquid interfaces play a vital role in regulating gas transport, as exemplified by the adaptive mechanisms of plant stomata and the liquid-lined alveoli, which enable efficient gas exchange through reversible opening and closing. These biological processes provide profound insights into the design of advanced gas control technologies. Inspired by these natural systems, liquid gating membranes have been developed utilizing capillary-stabilized liquids to achieve precise fluid regulation. These membranes offer unique advantages of rapid responses, stain resistance, and high energy efficiency. Particularly, they break through the limitations of traditional solid, porous membranes in gas transport. This perspective introduces bioinspired liquid gating gas valve membranes (LGVMs), emphasizing their opening/closing mechanism. It highlights how external stimuli can be exploited to enable advanced, multi-level gas control through active or passive regulation strategies. Diverse applications in gas flow regulation and selective gas transport are discussed. While challenges related to precise controllability, long-term stability, and scalable production persist, these advancements unlock significant opportunities for groundbreaking innovations across diverse fields, including gas purification, microfluidics, medical diagnostics, and energy harvesting technologies.

## 1. Introduction

**Figure 1:**
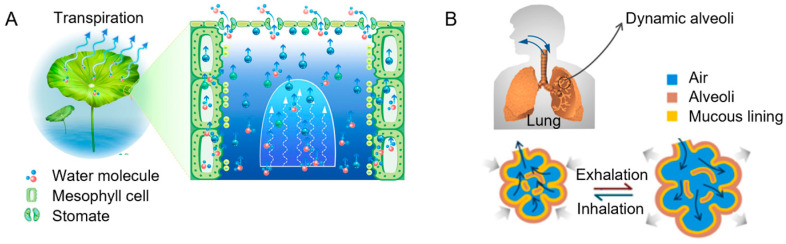
Biological valve structures in nature. (**A**) Gas exchange processes in plant stomata [9]. (**B**) Gas exchange processes in human alveoli [14].

In living organisms and biological processes, liquids play essential roles, offering valuable insights for advancing contemporary technologies. For instance, the liquid layer on the surface of Nepenthes pitcher plants aids in insect capture [1], the tear film on the eye provides a smooth refractive surface and protects against irritants [2,3], and synovial fluid in knee joints minimizes friction to facilitate smooth movement [4,5,6]. Beyond their function as lubricants, liquids also serve as key agents in regulating gas transport. In plants, for example, guard cells control gas exchange by modulating the opening and closing of stomata (Figure 1A), a process governed by water uptake or loss [7,8,9,10]. When guard cells absorb water, the thinner outer wall expands more significantly than the thicker inner wall, creating a gap between the cells and opening the stomata [11]. In contrast, water loss eliminates the gap, leading to stomatal closure. A similar phenomenon of liquid-based regulation of gas exchange also exists in animals (Figure 1B), particularly in the lungs—vital organs for respiration—which exhibit comparable functionality [12,13,14]. The alveoli contain tiny liquid-filled pores that dynamically respond to pressure changes, facilitating efficient gas exchange with the external environment. Inspired by these natural liquid functionalities, Hou et al. first proposed the concept of liquid gating mechanisms and developed liquid gating membranes [15]. They designed a reversible and reconfigurable gating system based on capillary-stabilized liquids, which effectively modulate fluid flow during transport processes. These membranes work by sealing pores with a complete liquid barrier in the closed state while creating a clean, liquid-lined pore in the open state. Notably, they possess remarkable features such as antifouling properties, rapid dynamic response, soft interfaces, structural plasticity, and defect-free performance [16,17,18]. These advancements underscore the potential of bioinspired liquid gating systems in addressing challenges across diverse technological applications [19,20,21,22].

Gas valves are indispensable in various industrial applications, particularly in areas such as petrochemicals, energy, and healthcare [23,24,25,26]. However, as the demands for efficient gas transport systems in these areas continue to rise, traditional gas valves face significant limitations, including the size of mechanical components, mechanical instability, low efficiency, susceptibility to clogging, and poor resistance to contamination. Conventional solid porous membranes, which typically feature pore sizes much larger than the gas molecules, are unable to regulate gas flow effectively through dynamic opening and closing [16]. In contrast, bioinspired liquid gating membranes, which utilize gating liquids as materials, offer dynamic and efficient gas flow regulation. Therefore, these membranes provide a solution to the shortcomings of traditional gas valves and overcome the limitations of conventional solid porous membranes in gas transport. We propose the term liquid gating gas valve membranes (LGVMs) to refer to such membranes specifically designed for gas valve applications. By leveraging the physicochemical design of functional interfaces, LGVMs provide expanded possibilities and unprecedented design flexibility for advanced gas control systems.

This perspective highlights the latest research advancements in LGVMs, with a particular focus on their design principles, mechanisms, and applications in gas flow rate regulation and selective gas transport. We explore the interaction between the solid–liquid interface and gas–liquid interface governing the opening and closing of gas valves and emphasize the use of external stimuli to achieve advanced, multi-level gas regulation in LGVMs through active or passive regulation strategies. We hope this perspective will accelerate the development of LGVMs, laying the foundation for innovative designs and wider applications in fields such as environmental sensing, pneumatic robots, biomedical devices, renewable energy solutions, and other advanced integrated systems.

## 2. Design Principle and Strategy of LGVMs

The design principle and strategy of LGVMs are highly important, integrating the complementary characteristics of liquid and solid materials, offering innovative functional advantages for gas valve technology. The core design concept of LGVMs revolves around utilizing a porous solid framework for structural support, with functional gating liquid filling the pores and harnessing capillary forces within the microchannels of the solid to achieve reversible gas flow control. The selection of solid materials necessitates careful consideration of factors such as porosity, pore size, pore geometry, surface roughness, and surface energy [16,27]. Materials with high porosity, excellent mechanical properties, and good chemical stability, such as metals, ceramics, polymers, and organic/inorganic hybrid membranes, are typically ideal candidates [28]. In addition, liquid materials exhibit unique flexibility in functional control due to their self-healing properties, defect-free nature, and dynamic responsiveness [29,30,31]. The selection of the gating liquid should be guided by the following three considerations: (1) the gating liquid must be infiltrated and stably adhered to the pores of the solid membranes; (2) the porous membrane has a higher affinity to the gating liquid than the transport fluid; (3) the gating liquid is not miscible with the transport fluid. To achieve these, the establishment of stable interfacial energy is indispensable [32]. The selection of liquid can be customized to meet specific application needs, considering factors such as surface tension, boiling point, solubility, viscosity, and conductivity. For instance, water-based, oil-based, ionic liquids, liquid metals, or functionalized solvents can be chosen based on these criteria [30,32,33,34].

The wetting behavior of the liquid on the solid surface is critical to the stability of LGVMs [28]. The contact angle serves as a key parameter for characterizing the wettability and non-wettability properties of the solid materials. A contact angle of 65° is generally regarded as the critical threshold between hydrophilicity and hydrophobicity. Below that value (hydrophilicity), the gating liquid easily wets the solid material, achieving stable gating functionality, while above this value (hydrophobicity), it becomes difficult for the gating liquid to wet the solid material, resulting in unstable gating functionality [35,36]. Furthermore, the adhesion between the porous solid membrane and the gating liquid is vital for maintaining the liquid gating functionality. Good wettability and certain adhesion can ensure that the gating liquid exists stably in the pores of the solid substrate. Additionally, the pore size of solid substrates also significantly impacts composite stability [32,37]. For LGVM systems, if the pores are too large, the composite materials combined gating liquid with porous solid may become unstable, leading to irreversibility in reconfiguration and an inability to perfectly fill the pores. Conversely, smaller pore sizes may lead to higher energy consumption in the application process. By integrating the structural support of solid materials with the dynamic response capabilities of liquids, LGVMs transform the conventional solid–gas interface into a solid–liquid–gas interface, constructing a defect-free, fast-response material system.

**Figure 2:**
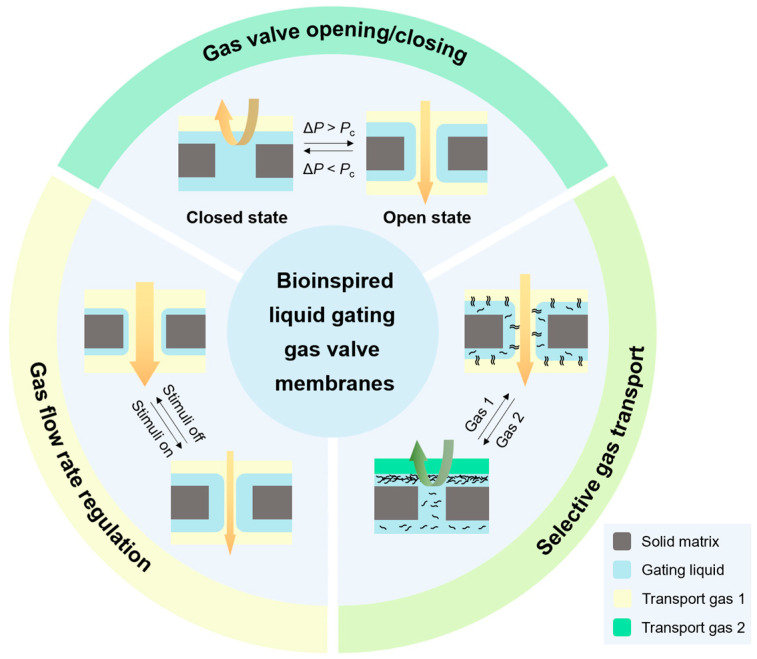
Overview of LGVMs for gas valve opening/closing, gas flow rate regulation, and selective gas transport, including a schematic of their typical response.

The gating mechanism of LGVMs utilizes capillary forces to stabilize liquids, functioning as pressure-driven, reversible, and reconfigurable valves [15,38]. In the closed state, the liquid fills and seals micro-scale pores, while in the open state, it forms a liquid-lined pore. When the applied pressure (∆*P*) is below the threshold pressure (*P*_c_) for gas transport (0 < ∆*P* < *P*_c_), the gas cannot penetrate the LGVMs. Once the applied pressure exceeds the critical value (∆*P* > *P*_c_), the LGVM permits gas transport. Upon pressure release, the liquid reverts to its original state, resealing the pores (Figure 2). The threshold pressure is directly related to the surface tension (*γ*) of the gating liquid and the pore size (*D*) of the porous solid.

*P*_c_ = 4*γ*/*D* (1)

To achieve more multifunctional gas delivery and release, it is helpful to design the LGVMs for active or passive stimulus response in enhancing performance. With the continuous exploration of responsive design for LGVMs, strategies based on various external stimuli, such as light [38], acoustic field [39], electricity [40], magnetism [41], heat [42], stress [43] and chemical stimulation [44], are increasingly being developed. LGVMs are capable of conducting gas valve switch control, gas flow rate regulation, and selective gas transport via these strategies (Figure 2).

## 3. Progress of LGVMs

LGVMs offer an adaptable switching mechanism for regulating gas transport. For instance, Hou et al. employed capillary-stabilized liquids as reversible, reconfigurable gates, where the liquid fills and seals the pores, thereby blocking gas flow in the closed state [15]. When enough pressure is applied, the valve opens and the gas passes through, which can, simply, achieve the control of gas flow.

**Figure 3:**
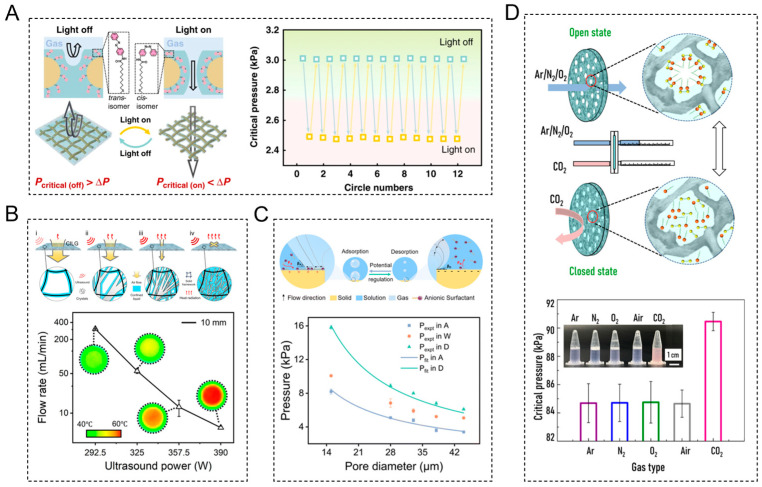
(**A**) Gas valve on/off control by light response [38]. (**B**) Gas flow rate regulation by acoustic response [45]. (**C**) Bubble size regulation by electric field response [40]. (**D**) Selective gas transport by chemical response [44].

To enhance the accuracy, interfacial transmission efficiency, and remote operability of gas valves, there has been growing development regarding responsive LGVMs. For instance, Chen et al. incorporated azobenzene-based molecular photoswitches (Table 1) into the solid substrate of a stainless-steel membrane to create a light-responsive and corrosion-resistant gas valve (Figure 3A) [42]. The interaction between the functional liquid and the membrane can be modulated by ultraviolet (UV) light stimulation. Without UV light, the azobenzene molecules show a trans configuration, exhibiting strong interactions with the gating liquid, which prevents gas transport. Upon UV irradiation, the azobenzene molecular photoswitches undergo trans-to-cis photoisomerization, significantly reducing the solid–liquid interaction, which enables gas transport and transitions the system to an open state. It can serve as an effective non-thermal, light-activated gas valve, enabling dynamic and regulated control of gas flow within a specified region. It provides an active way by applying external field stimulation to reduce the threshold pressure and make the gas flow pass through. In contrast, another study introduced a passive way of making photosensitive molecules into the gating liquid [37]. The light-responsive gating liquid was prepared by dissolving the azobenzene-derived photoresponsive surfactant molecule AzoC_8_F_15_ in N, N-dimethylacetamide. This functional liquid was then integrated with a nylon porous substrate to form a bioinspired photoresponsive liquid gating membrane. Upon exposure to UV light, AzoC_8_F_15_ molecules experience a trans-to-cis isomerization process, which leads to an increase in surface tension, and then an increase in critical pressure, which impedes gas from passing through the valve. It has the opportunity to be used in the precise and contactless control of microfluidics. Moreover, the acoustic field can also be employed as a passive control mode. Liu et al. developed a gas valve system based on a non-Newtonian fluid gating membrane, utilizing the shear-thickening behavior of corn starch suspensions and copper foam [39]. When subjected to acoustic field stimulation, the friction and contact of the particles in corn starch suspensions increase the pressure threshold, effectively blocking gas transport. It could be leveraged for the development of integrated smart materials tailored for operation in complex and extreme environments, including the transport of hazardous and explosive gases.

These responsive LGVMs possess the capacity not only to effectuate favorable switching control but also to perform highly flexible modulation of gas flow rate. For example, Han et al. designed a crystallization-induced liquid gate (CILG) using a supersaturated solution (sodium acetate trihydrate) as the functional liquid and a stainless-steel mesh as a porous membrane [45]. Ultrasound stimulation reduces the energy barrier between the supercooling state and the crystalline state. The newly formed crystals by ultrasound effectively reduce the pore size of the solid matrix, modulating the threshold pressure required to pass through the CILG. By adjusting the ultrasound power, the density of crystal growth can be controlled, which in turn regulates the pore size and transmembrane pressure (Figure 3B). This straightforward method provides a flexible strategy for gas flow rate control, holding significant promise for applications in smart gas valves, environmental management, and energy development.

In addition to controlling the gas flow rate, LGVMs can also efficiently regulate bubble behavior, offering neoteric solutions for microfluidic systems. Zhang et al. employed an interfacial polymerization approach to deposit sodium dodecyl benzenesulfonate doped polypyrrole (PPy) on a stainless-steel mesh to construct an electrochemical liquid-based system (ELBS) [46]. By applying an electric field to modulate the water–phase interface, they altered the redox state of the PPy coating, thus adjusting the hydrophilicity of the solid surface and finally influencing microbubble formation and growth. In the reduced state, increased hydrophilicity facilitates the generation of smaller bubbles, which enhances the gas–liquid contact area, improving the efficiency of mass transfer and particulate capture. The ELBS system achieved an air purification efficiency as high as 99.6%. This research explores the dissolution, diffusion, and absorption mechanisms of gaseous pollutants at liquid-based interfaces and demonstrates how liquid materials can be employed to modulate microbubbles via an electrochemical method. This control enhances mass transfer at the three-phase interface for efficient removal of harmful gases and particles from the air, with a crucial role in odor removal and bacterial elimination. Additionally, an aqueous solution of sodium dodecyl sulfate is used as the gating liquid, while a microporous stainless-steel membrane coated with gold served as the porous solid material. By adjusting the applied voltage, the adsorption and desorption of anionic surfactants on the gold-coated surface can be controlled, enabling more flexible and rapid microbubble size regulation [40]. Under more negative potentials, anionic surfactants desorb from the surface due to electrostatic repulsion which decreases the contact angle and facilitates microbubble formation (Figure 3C). This method of dynamically adjusting the microbubbles through an electric field offers more possibilities for the LGVMs.

Beyond regulating the transport of a specific gas, LGVMs can also selectively respond to different gas species. For example, Lei et al. developed a CO_2_-responsive gating liquid by assembling amphiphilic molecules (poly(propylene glycol) bis(2aminopropyl ether) and oleic acid) in an aqueous solution [44]. This gating liquid was then integrated with a nylon porous membrane to form a protonation-induced liquid gating system (Figure 3D). The underlying mechanism of this system is based on CO_2_-induced protonation, which induces the rearrangement of the interfacial amphiphilic molecules, thereby modulating the threshold pressure of the system. The system exhibits unique selective responsiveness to CO_2_: when CO_2_ is the transport gas, the valve remains closed, while it opens when the gas is N_2_, O_2_, or Ar. Furthermore, the system is capable of self-adaptive regulation based on CO_2_ concentration: at lower concentrations, the surface tension of the gating liquid decreases, causing the valve to open; at higher concentrations, enhanced protonation increases the critical pressure, leading to valve closure. These selective flow control mechanisms possess considerable potential in applications such as gas separation, environmental monitoring, and industrial emissions management.

## 4. Challenges and Outlook

Liquid gating gas valve membranes address the limitations of traditional mechanical gas valves, such as bulkiness and inflexibility, and overcome the inability of conventional solid porous membranes to regulate gas transport [42,43,44,45]. These membranes also provide smooth, defect-free surfaces. Currently, LGVMs have made significant progress in gas transport control, gas flow rate regulation, and selective gas transport through their responsiveness to external stimuli. However, several challenges remain in practical applications, particularly in achieving precise controllability, long-term stability, and scalable fabrication. Gas regulation can be inconsistent under fluctuating environmental conditions due to non-linear responses in the liquid interface. Issues like liquid evaporation, leakage, contamination, and mechanical degradation of porous membranes further limit durability. Additionally, current materials often face trade-offs between performance, environmental impact, and cost, while the energy demands of external stimuli-based control mechanisms can hinder efficiency. Scalable production methods and seamless integration into existing systems also pose significant challenges.

With the rapid development of materials science, novel solid and liquid materials are emerging. Innovative material designs, such as robust, environmentally friendly liquids and hybrid membranes with hierarchical structures, can enhance stability and performance. In parallel, biomimetic designs inspired by diverse natural systems could enable more advanced and responsive gas control systems. For instance, integrating dual-liquid systems or multifunctional layers could provide selective and adaptive gas transport capabilities. Further, incorporating catalytic functionalities or sensors into LGVMs could transform them into multifunctional components for gas purification, conversion, and monitoring [47]. With scalable fabrication techniques, such as 3D printing and interdisciplinary collaborations incorporating cutting-edge achievements in artificial intelligence, computational modeling, and nanotechnology, efforts can focus on optimizing membrane interface design and improving material performance, which will be essential to transition LGVMs from prototypes to industrial applications. These advancements are promising to drive significant progress in a variety of fields, including environmental governance, energy systems, biomedical engineering, bioengineering, and adaptive robotics, fostering the development of more efficient, sustainable, and multifunctional technologies for future applications.

## Figures and Tables

**Table 1:** Summary of reported responsive LGVMs, including the external stimuli, the responsive materials, and the applications.

External Stimuli	Responsive Materials		Applications
	Solid Porous Membranes	Gating Liquids	
Ultraviolet [42]	Azobenzene-based stainless-steel mesh	Krytox 103	Positional flow control
Ultraviolet [37]	Nylon porous substrate	Photoresponsive surfactant molecule	Precise and contactless control of microfluidics
Acoustic field [39]	Copper foam	Corn starch suspension	Transport of hazardous and explosive gases
Ultrasound stimulation [45]	Stainless-steel mesh	Sodium acetate trihydrate	Infrared-monitored flow-regulating valve
Electric field [46]	Stainless-steel mesh with sodium dodecyl benzene-sulfonate	LiClO_4_ aqueous solution	Air purification
Chemical stimulation [44]	Nylon porous membrane	Amphiphilic molecule	Gas separation, CO_2_ capture

## Data Availability

Not applicable.
